# Characterization of a murine mixed neuron-glia model and cellular responses to regulatory T cell-derived factors

**DOI:** 10.1186/s13041-018-0367-6

**Published:** 2018-05-02

**Authors:** Marie Dittmer, Andrew Young, Thomas O’Hagan, George Eleftheriadis, Peter Bankhead, Yvonne Dombrowski, Reinhold J. Medina, Denise C. Fitzgerald

**Affiliations:** 1 Centre for Experimental Medicine, School of Medicine, Dentistry and Biomedical Science, Queen’s University Belfast, Belfast, Northern Ireland, UK; 2 Centre for Cancer Research and Cell Biology, School of Medicine, Dentistry and Biomedical Science, Queen’s University Belfast, Belfast, Northern Ireland, UK

**Keywords:** Murine mixed glia, OPC, Oligodendrocyte, Treg

## Abstract

One of the unmet clinical needs in demyelinating diseases such as Multiple Sclerosis (MS) is to provide therapies that actively enhance the process of myelin regeneration (remyelination) in the central nervous system (CNS). Oligodendrocytes, the myelinating cells of the CNS, play a central role in remyelination and originate from oligodendrocyte progenitor cells (OPCs). We recently showed that depletion of regulatory T cells (Treg) impairs remyelination in vivo, and that Treg-secreted factors directly enhance oligodendrocyte differentiation. Here we aim to further characterize the dynamics of Treg-enhanced oligodendrocyte differentiation as well as elucidate the cellular components of a murine mixed neuron-glia model.

Murine mixed neuron-glia cultures were generated from P2–7 C57BL/6 mice and characterized for percentage of neuronal and glial cell populations prior to treatment at 7 days in vitro (div) as well as after treatment with Treg-conditioned media at multiple timepoints up to 12 div.

Mixed neuron-glia cultures consisted of approximately 30% oligodendroglial lineage cells, 20% neurons and 10% microglia. Furthermore, a full layer of astrocytes, that could not be quantified, was present. Treatment with Treg-conditioned media enhanced the proportion of MBP^+^ oligodendrocytes and decreased the proportion of PDGFRα^+^ OPCs, but did not affect OPC proliferation or survival. Treg-enhanced oligodendrocyte differentiation was not caused by Treg polarizing factors, was dependent on the number of activation cycles Treg underwent and was robustly achieved by using 5% conditioned media.

These studies provide in-depth characterization of a murine mixed neuron-glia model as well as further insights into the dynamics of Treg-enhanced oligodendrocyte differentiation.

**Electronic supplementary material:**

The online version of this article (10.1186/s13041-018-0367-6) contains supplementary material, which is available to authorized users.

## Introduction

Multiple Sclerosis is one of a number of demyelinating diseases of the central nervous system (CNS) yet there are still no therapies that actively enhance the process of myelin regeneration (remyelination). Uncovering the mechanisms that govern remyelination and developing novel remyelinating treatments could prove highly beneficial to patients’ quality of life [1, 2]. For efficient central nervous system remyelination to occur, oligodendrocyte progenitor cells (OPCs) have to successfully migrate, survive, proliferate, differentiate, mature and wrap myelin sheath around axons. While one study showed that OPCs of sufficient numbers were present in about 80% of chronic demyelinating lesions of early MS patients [3], another study reported lesions with insufficient OPC numbers, indicating OPC migration or proliferation as limiting factors in impaired remyelination [4]. However, enhanced OPC proliferation and therefore available OPC numbers in lesions does not appear to influence remyelination in a model of lysolecithin-induced demyelination [5]. Therefore, a key bottle-neck in remyelination appears to be oligodendrocyte differentiation and subsequent remyelination [3, 6, 7]. Currently there are no therapies to enhance oligodendrocyte differentiation, highlighting the need for appropriate, well-characterized and efficient models to uncover and develop novel treatments.

A wide variety of experimental approaches have been employed to study CNS (re)myelination. These approaches include toxin-, viral- and immune-mediated in vivo models of demyelinating disease, which are useful for studying the complexity of de- and remyelination in vivo. However, to study the cellular mechanisms governing these processes, simpler models that permit more specific investigations at cellular and molecular levels are necessary*.* Ex vivo brain slice models can be used to study developmental myelination as well as demyelination and subsequent remyelination [8]. These models mirror the complexity and relative cytoarchitecture of CNS tissues and due to a lack of connection to the circulation, specific neural tissue responses can be studied without the influence of infiltrating leukocytes. Finally, there are in vitro models that facilitate the investigation of molecular and cellular mechanisms underlying regeneration, such as the mixed glia model that comprises cell types commonly found in the CNS but not in typical functional architecture. In vitro models are therefore valuable tools to study molecular mechanisms and cell-specific effects. While mixed glia models are commonly used, surprisingly little is published regarding model characterization and even the name mixed glial culture can be considered a misnomer, as despite this name, these cultures generally also contain other CNS-resident cell types including neurons. Therefore, we sought to provide an in-depth characterization of a murine mixed neuron-glia in vitro model.

Recently, a growing body of research into the regenerative properties of regulatory T cells (Treg) in multiple tissues including the lung, skin, spinal cord, muscle and myocardium has emerged [9–15]. We showed for the first time that murine Treg play a crucial role in myelin generation and regeneration and can secrete factors capable of directly enhancing oligodendrocyte differentiation [16]. The Karimi-Abdolrezaee group showed that Neuregulin-1 promotes remyelination in lysolecithin-induced demyelination and they found a corresponding increase of Treg in lesions of Neuregulin-1 treated animals 14 days post-lesioning [17]. In this study, we sought to characterize a murine mixed neuron-glia model through an investigative study of Treg influence on oligodendrocyte development.

The reductionist murine mixed neuron-glia model is a useful tool to study basic immune cell responses in the context of CNS cells. While devoid of peripherally-derived infiltrating leukocytes, this model strikes a balance between the tissue complexity of ex vivo brain slice models and pure OPC models, which completely lack the diversity of CNS cells. Therefore, the murine mixed neuron-glia model is ideal to study fundamental cellular processes underlying neuro-immune interactions in the CNS. In this study, we provide in-depth characterization of a murine mixed neuron-glia model as well as detailed methods and characterization of experimental conditions, including media type, different concentrations and timecourses that facilitate Treg-enhanced oligodendrocyte differentiation. These studies are critical to understand the nuances of Treg-mediated regulation of oligodendrocyte development. This study can therefore aid the design of future studies investigating the effects of other (immune) cell subsets on CNS cell populations.

## Materials and methods

### Animals

Mice were housed under standard laboratory conditions (12/12 h light/dark cycle with a room temperature of 21 °C, humidity of 50% and water and food available *ad libitum*). C57BL/6 mice were bred in-house or bought from Charles River Laboratories and maintained in-house. PLP-eGFP mice were a kind gift from Prof. Wendy Macklin, Cleveland Clinic Foundation [18] and maintained in-house. Male and female C57BL/6 mice aged 2 to 9 postnatal days were used for mixed glial and pure OPC cultures. Spleens from either all male or all female C57BL/6 mice aged 6 to 12 weeks were used for T cell cultures. All animal maintenance and experiments were in compliance with the UK Home Office and approved by the Queen’s University Belfast Animal Welfare and Ethical Review Body (AWERB).

### T cell culture, polarization and conditioned-media generation

Spleens from C57BL/6 mice aged 6–12 weeks were extracted, passed through a 70 μm strainer and washed with Phosphate Buffered Saline (PBS). Total or naïve (CD62L^+^CD44^−^) CD4^+^ T cells were purified using the EasySep Mouse CD4^+^ T cell isolation kit (Stemcell Technologies Inc.) as per manufacturer’s instructions. In general, for total CD4^+^ T cell isolation, splenocytes were counted and resuspended to 1 × 10^8^ cells/ml in purification buffer containing 2% Foetal Bovine Serum (FBS) and 1 mM EDTA in PBS. Next, normal rat serum (50 μl/ml) as well as EasySep mouse CD4^+^ T cell isolation cocktail (50 μl/ml) were added, mixed and incubated for 10 min at room temperature (RT). Afterwards, EasySep Streptavidin RapidSpheres (75 μl/ml) were added, mixed and incubated for 2.5 min at RT. The suspension was brought up to a total volume of 2.5 ml by adding purification buffer and the tubes containing the suspension were placed into the EasySep magnet for a further 2.5 min at RT. The purified CD4^+^ cells were transferred into a new sterile tube. Purity was assessed via flow cytometry and samples only further used if purity was above 90%. At this point CD4^+^ T cells were counted, resuspended in RPMI media (Invitrogen, 10% FBS, 1% sodium pyruvate, 1% HEPES, 1% non-essential amino acids, 1% Penicillin/Streptomycin, 1% L-Glutamine, 50 nM β-Mercaptoethanol) and plated at a density of 1 × 10^6^ cells/ml in 1 ml volume per well of a tissue culture-treated 24-well plate. CD4^+^ T cells were activated through the addition of anti-CD3 (1 μg/ml, clone 145-2C11, eBioscience) and anti-CD28 (1 μg/ml, clone 37.5, eBioscience). Treg were polarized in vitro through addition of murine IL-2 (10 or 20 ng/ml, eBioscience), human TGF-β (2 or 4 ng/ml, eBioscience) and anti-IFN-γ (10 μg/ml, BioXCell). Conditioned media from these cultures were harvested after 3 days and stored at − 80 °C. T cells were washed, counted and polarization efficiency was assessed via flow cytometry and samples only used in further experiments if Treg polarization efficiency was above 80% as determined by FoxP3 expression. Cells were replated at 1 × 10^6^ cells/ml in 1 ml volume per well of a 24-well plate in different media (brain slice media (BSM) (MEM, 25% EBSS, 25% heat-inactivated horse serum, 1% Penicillin/Streptomycin, 1% GlutaMAX, 0.65% D-glucose) or X-VIVO™ 15 (Lonza)) and again activated and polarized as before. Conditioned media were harvested on the third day of the first, second or third activation, spun down at 300 g for 5 min and stored at − 80 °C. Polarization efficiency was checked after each activation and samples only further used if Treg polarization efficiency was above 80% as determined by FoxP3 expression.

### Flow cytometry

For flow cytometric analysis up to 1 × 10^6^ cells per tube were stained with surface antibodies at 1:1000 (CD45 (clone 104, eBioscience), CD3 (clone 17A2, eBioscience), CD4 (clone GK1.5, eBioscience)) for 20 min at RT. Cells were then fixed with 100 μl Fix&Perm Reagent A (Invitrogen) for 15 min at RT and then stained with 1 μl of intracellular antibodies (FoxP3 (clone FJK-16 s, eBioscience)) in 100 μl Fix&Perm Reagent B (Invitrogen) overnight at 4 °C. Fixed and permeabilized unstained cells were used as a negative control, single color surface stained cells or UltraComp beads (eBioscience) were used for compensation and Fluorescence Minus One (FMO), meaning cells that were identically stained as samples, but were lacking one staining antibody at a time, were used to control for multiple fluorochrome staining particularly for intracellular targets. Cells and controls were analyzed using a BD FACS Canto II cytometer and data were subsequently analyzed using FlowJo software (Treestar Inc.).

### Mixed neuron-glia cultures

Mixed neuron-glia cells were generated from P2–7 C57BL/6 mouse pups according to the protocol of the Neural Tissue Dissociation Kit (P) (Miltenyi Biotec). In general, brains were dissected, cerebellum, olfactory bulbs and meninges were removed and tissue was cut into small pieces. Up to 2.5 brains per 15 ml falcon tube were papain enzyme digested with enzyme mix 1, consisting of 50 μl enzyme P and 1900 μl buffer X. The enzyme mix was incubated with the dissociated tissue for 15 min at 37 °C on a rotator. After addition of enzyme mix 2, consisting of 10 μl enzyme A and 20 μl of buffer Y, tissues were very slowly dissociated mechanically using pasteur as well as P1000 pipettes interspaced with two more 10 min incubations on a rotator at 37 °C. The suspension was then filtered through a 40 μm strainer and cells pelleted at RT for 10 min at 300 rcf prior to resuspension in complete DMEM media (cDMEM, 10% endotoxin-low FBS, 1% P/S, 1% L-glutamine). Black, flat-bottomed, TC-treated, 96-well imaging plates (BD Falcon) were coated with 50 μl per well of poly-L-lysine (10 μg/ml, Sigma) for at least 4 h at RT. Cells were plated on poly-L-lysine-coated plates at a density of 5 × 10^5^ cells/ml and 200 μl volume per well and cultures were maintained at 37 °C, 5% CO2. Cells were cultured for 5 days in cDMEM supplemented with human PDGFαα (10 ng/ml, Peprotech) during which time an astrocyte monolayer formed. Cells were cultured for a further 2 days in complete Neural Medium (cNM, 2% MACS NeuroBrew-21, 1% P/S, 1% L-glutamine) supplemented with PDGFαα (10 ng/ml, Peprotech) for initial OPC expansion. At day 7 in culture, PDGFαα supplementation was stopped to allow oligodendrocyte differentiation and cells were stimulated as described in the appropriate figures for up to 5 days.

### Pure OPC cultures

Pure OPC cultures were generated from P5–9 C57BL/6 mouse pups according to a protocol adapted from Rena Hesse et al. [19]. Brains were dissected, cerebellum, olfactory bulbs and meninges were removed and tissue was cut into small pieces. One to three brains per 10 cm dish were papain enzyme-digested with 165 U papain (Worthington) in 10 ml papain buffer (10% EBSS, 3.6 mg/ml glucose, 26 mM NaHCO_3_, 1 mM MgSO_4_, 2 mM EGTA, 0.2 mg/ml L-cysteine, 40 mg/l DNase I) for 5 min at 37 °C. Tissues were mechanically dissociated and step by step triturated in high inhibition buffer (10% EBSS, 3.6 mg/ml glucose, 26 mM NaHCO_3_, 10 mg/ml ovomucoid trypsin inhibitor, 10 mg/ml BSA, 33.3 mg/ml DNase I) and then low inhibition buffer (10% EBSS, 3.6 mg/ml glucose, 26 mM NaHCO_3_, 1.3 mg/ml ovomucoid trypsin inhibitor, 1.3 mg/ml BSA, 17.4 mg/ml DNase I). The cell suspension was spun down at 200 rcf for 10 min at RT and the visible cell swirl was collected and resuspended in DPBS (Invitrogen) containing 0.2% BSA and 5 mg/l insulin. The remaining cell suspension was again spun down at 300 rcf for 10 min at RT and the visible cell swirl was again collected into the same panning buffer-containing tube. Cells were then negatively panned for 15 min at RT using Bandeira Griffonia Simplicifolia Lectin 1 (BSL1) (10 cm dish precoated overnight at 4 °C with 0.2% BSL1 in DPBS, Vector Laboratories) to remove microglia. Afterwards pure OPCs were positively selected by panning for PDGFRα (10 cm dish pre-coated overnight at 4 °C with 0.3% goat anti-rat IgG (Invitrogen) and then coated with 0.15% rat anti-PDGFRα (clone 38 APA5, Biolegend) for more than 4 h at RT). Pure OPCs were scraped off after vigorous washing, plated on poly-L-lysine-coated T75 flasks (10 μg/ml, 10 ml per flask, Sigma) at 1–3 brains per flask in OPC medium (DMEM with Glutamax, 0.04% B27 supplement, 100 ng/ml apo-transferrin, 100 ng/ml BSA, 62.5 ng/l progesterone, 16 ng/ml putrescine, 40 ng/l sodium selenite, 1% P/S, 60 mg/l N-acetyl-cysteine, 5 mg/l insulin, 0.1% Trace elements B, 1 ng/l biotin, 2 mg/l forskolin) and maintained at 37 °C, 5% CO2. Medium was changed every 3 days and cells were supplemented daily with human PDGFαα (10 ng/ml, Peprotech) and human NT3 (5 ng/ml, Peprotech). Black, flat-bottomed, TC-treated, 96-well imaging plates (BD Falcon) were coated with 50 μl per well of poly-L-lysine (10 μg/ml, Sigma) overnight at 4 °C and on the next day with 50 μl per well of laminin (10 μg/ml, Sigma) for at least one hour at 37 °C. After 7 days of proliferation, cells were transferred onto poly-L-lysine- and laminin-coated plates at a density of 2 × 10^5^ cells/ml and 200 μl volume per well and maintained for a further 2 days under proliferating conditions. At day 9 in culture, PDGFαα and NT3 supplementation were stopped to allow oligodendrocyte differentiation and cells were stimulated as described in the appropriate figures for up to 3 days. Purity was assessed in random cultures at 3 to 5 days after setup and was usually over 93% as determined by percentage Olig2^+^ cells of total DAPI^+^ cells.

### LDH assay

To assay potential cell death in mixed neuron-glia cultures, conditioned media were harvested and stored at − 20 °C prior to testing. Lactate dehydrogenase (LDH) activity, as a surrogate indicator of cell death, was measured in conditioned media by a Cytotoxicity Detection Kit (LDH) (Roche) as per manufacturer’s instructions. In general, 100 μl of LDH reaction mixture (1:45 Catalyst:Dye ratio) was added to 100 μl of mixed neuron-glia conditioned media (*n* = 6 biological replicates per timepoint) in clear, flat-bottomed 96 well plates and incubated in the dark for 30 min at RT. A background level of LDH/substrate catalytic activity was determined by assaying medium-only control, while a maximal cell death control was measured by testing conditioned media from mixed neuronal-glia cultures treated with 10% DMSO. After incubation, samples were assayed using an absorbance value of 491 nm, with a reference absorbance of 600 nm, using an Epoch Microplate Spectrophotometer (BioTek). Optical density (OD) values were calculated by subtracting the 600 nm reference value from the 491 nm value, followed by subtracting the medium-only control value. To ascertain a surrogate readout for percentage of total cell death OD values were divided by the maximum cell death control (mixed neuron-glia treated with 10% DMSO).

### Immunofluorescence

Mixed neuron-glia cultures were washed once in PBS prior to fixation, whereas pure OPC cultures were fixed directly. Cell cultures were fixed in 4% paraformaldehyde (pH 7.4) (Sigma) for 15–20 min at RT. After blocking in 10% normal goat serum (Vector Laboratories) with 0.1% Triton-X-100 in PBS for 1 h, cells were incubated with primary antibodies (anti-PDGFRα (clone APA5, 1:200, Biolegend), anti-Olig2 (Millipore), anti-MBP (clone 12, 1:1000, Millipore), anti-MAP2 (clone AP-20, 1:200, Abcam), anti-NF200 (clone RT97, 1:200, Millipore), anti-APC (clone CC-1, 1:100, Abcam), anti-Ki67 (clone SolA15, 1:200, eBioscience), anti-Iba1 (1:600, Wako), anti-Nkx2.2 (clone 74.5A5, 1:200, DSHB), anti-CD11b (clone M1/70, 1:200, eBioscience), anti-GFAP (1:200, Dako), anti-CD45 (clone 104, 1:200, eBioscience), anti-PDGFRβ (clone 28E1, 1:100, Cell signaling), anti-CD31 (clone H-3, 1:100, Santa Cruz), anti-A2B5 (clone A2B5–105, 1:200, Millipore), anti-NG2 (clone 132.39, 1:200, Millipore), anti-Sox2 (clone 20G5, 1:100, Abcam), anti-Oct4 (1:100, Abcam), anti-Sox1 (1:100, Abcam), anti-O4 (clone 81, 1:200, Millipore)) in 2% normal goat serum with 0.001% Triton-X-100 in PBS overnight at 4 °C and in secondary antibodies (AF488 and AF594, 1:1000, Invitrogen) for 1 h at RT. Cells were counterstained with DAPI for 5 min at RT. For live/dead staining unfixed cells were stained using the LIVE/DEAD© Viability/Cytotoxicity kit, for mammalian cells (Invitrogen). Cells were stained with 0.2 μM calcein and 2 μM Eth-D1 for 15 min at 37 °C and directly imaged.

### Image analysis and data processing

Immunofluorescence was detected using an EVOS FL, EVOS FL Auto or CellInsight CX4 microscope at 10× or 40× magnification and *n* = 3 to 6 wells per condition. Four to 20 distinct field of view (FOV) images per well were analyzed to generate mean values for each well. Counts of oligodendrocyte lineage marker Olig2, OPC marker PDGFRα, differentiated oligodendrocyte marker MBP, proliferation marker Ki67, neuronal marker MAP2 and microglial marker CD11b positive cells were performed manually. For Fig. 6 counts of Olig2^+^Ki67^+^ cells were determined with the cell count tool of IMARIS software. Automated counting via IMARIS software (Bitplane) was validated by manual blinded Olig2^+^ cell counts. MBP^+^ areas were determined using a fully automated custom FIJI plugin [20], that was developed in-house (available upon reasonable request from the corresponding author). Briefly, MBP^+^ areas were determined from the number of above-threshold pixels after applying a small median filter (radius = 1.5 pixels), followed by subtracting a background image generated by the ‘subtract background’ command of ImageJ (sliding paraboloid option, radius = 100 pixels). For each plate the threshold was determined and the same threshold was applied to all analyzed images within each experiment. While MBP^+^ area readouts are a more accurate measure of oligodendrocyte myelin protein elaboration, the use of MBP^+^ area readouts as a surrogate measure of Treg-enhanced oligodendrocyte differentiation was validated by manual blinded MBP^+^ cell counts.

### Statistical analysis

Datasets were tested for statistical significance using unpaired, two-tailed, Student’s t tests for parametric data and Mann-Whitney tests for non-parametric data. Normality of datasets was determined using the KS normality test. All statistical analysis and graphing were performed using GraphPad Prism software.

## Results

### Characterization of mixed neuron-glia culture composition

To study the effects of Treg on OPCs in vitro, a model containing a good proportion of OPCs was required. In our hands, the murine mixed glial model, despite its name, does not only contain populations of all main glial cell types, but also a sizable population of MAP2^+^ neurons, thereby providing an in vitro model containing all the main cell types commonly found in the CNS. Therefore, we sought to carefully characterize the different cell populations present in this mixed neuron-glia model.

**Fig. 1:**
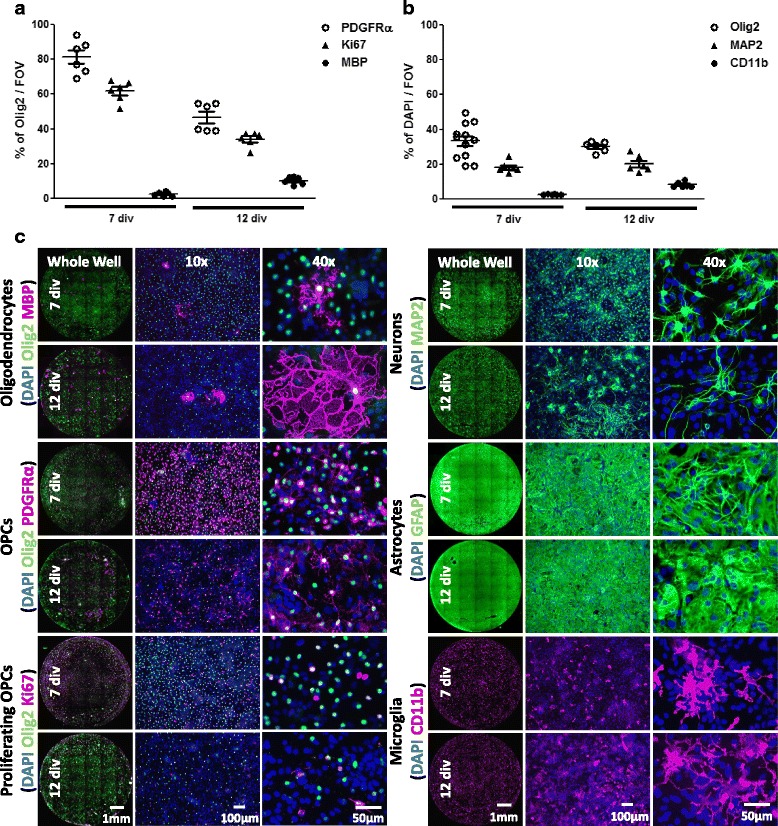
Oligodendroglial lineage cell composition in mixed neuron-glia cultures. Immunofluorescence analysis (**a**) of Olig2^+^ oligodendroglial cell population for percentage of OPCs (PDGFRα^+^), oligodendrocytes (MBP^+^) and proliferating OPCs (Ki67^+^) and (**b**) of DAPI^+^ cells for percentage of oligodendroglial lineage cells (Olig2^+^), neurons (MAP2^+^) and microglia (CD11b^+^) in mixed neuron-glia cultures at 7 or 12 div, *n* = 6 to 12 wells, representative of two independent experiments. Data are from 4 different experiments depending on timepoint and marker. FOV = field of view = 1 mm^2^. (**c**) Representative images of mixed neuron-glia cultures stained for oligodendrocytes (Olig2^+^MBP^+^), proliferating OPCs (Olig2^+^Ki67^+^), OPCs (Olig2^+^PDGFRα^+^), neurons (MAP2^+^), astrocytes (GFAP^+^), microglia (CD11b^+^) and total cells (DAPI^+^) at 7 and 12 div

To generate cultures that contained a sufficient number of OPCs prior to treatment, murine mixed neuron-glia cultures were expanded for 7 days in vitro (div) in the presence of PDGFαα and stained for the oligodendroglial lineage marker Olig2, together with a marker of OPCs (platelet-derived growth factor receptor α (PDGFRα)), proliferating cells (Ki67) and oligodendrocytes (myelin basic protein (MBP)). Indeed, at 7 div the Olig2^+^ oligodendroglial lineage cell compartment predominantly consisted of OPCs (approximately 80% PDGFRα^+^), with a high proportion of proliferating OPCs (approximately 60% Ki67^+^) and only a small percentage of differentiated MBP^+^ oligodendrocytes (< 5%) (see Fig. 1a, c). To further characterize the composition of mixed neuron-glia cultures at 7 div, cultures were additionally stained for markers of astrocytes (Glial Fibrillary Acidic Protein (GFAP)), microglia (CD11b) and neurons (Microtubule Associated Protein 2 (MAP2)). Based on total DAPI^+^ cell, mixed glial cultures contained approximately 30% of Olig2^+^ oligodendrocyte lineage cells, approximately 20% of neurons, approximately 5% of microglia and a full layer of astrocytes, that was not readily quantifiable via cell counting (see Fig. 1b, c). Based on the observed astrocytic layer it is likely that astrocytes accounted for about 45% of the remaining cells present in mixed neuron-glia cultures. As mixed cultures were generated from pups with ages ranging from 2 to 7 days, the individual cell percentages did vary slightly between different cultures (approximately +/− 5–10% depending on the population), though the percentages reported were the most representative. The existence of other potential cell populations within mixed neuron-glia cultures was also further investigated. At 7 div Nestin^+^Sox1^+^ neural progenitor cells (NPCs) could not be detected and there was only a very small population of CD31^+^ endothelial cells (see Additional file 1: Figure S1). PDGFRβ^+^ pericytes were also undetectable at 7 div (see Additional file 1: Figure S1). Additional markers used to characterize these mixed neuron-glia cultures were CD45^+^Iba1^+^ for microglia, NF200^+^ for neurons, A2B5^+^ and Nkx2.2^+^ for OPCs, O4^+^ for pre-oligodendrocytes as well as APC^+^ and PLP^+^ for oligodendrocytes (see Additional file 1: Figure S1).

After 7 div PDGFαα was withdrawn to allow natural oligodendrocyte differentiation to occur for 5 days. At 12 div the oligodendroglial lineage cell compartment had changed considerably. The percentage of PDGFRα^+^ OPCs decreased by approximately 30% (approximately from 80% at 7 div to 50% at 12 div), whereas the percentage of MBP^+^ oligodendrocytes proportionally increased by approximately 10% (approximately from < 5% at 7 div to 10% at 12 div) while the percentage of Olig2^+^Ki67^+^ proliferating OPCs decreased by approximately 20% (approximately from 60% at 7 div to 40% at 12 div) (see Fig. 1a, c). Overall percentages in neuronal and glial compartments remained largely unchanged during oligodendrocyte differentiation (see Fig. 1b, c), although Olig2 staining intensity was markedly dimmer at the later timepoint, likely caused by a down-regulation of the transcription factor Olig2 during oligodendrocyte differentiation. Interestingly, the appearance of MAP2^+^ neurons changed from single cells with more ramified morphology to largely bipolar shaped cell clusters that were oriented in the same direction (see Fig. 1b, c).

### Treg-conditioned media increase the proportion of differentiated oligodendrocytes

**Fig. 2:**
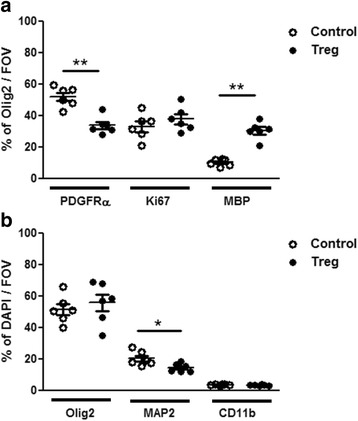
Treg enhance OPC differentiation and reduce neuronal percentages in mixed neuron-glia cultures. Mixed neuron-glia cultures were treated with 5% Treg-conditioned media or matched medium control for 5 days. Immunofluorescence analysis (**a**) of Olig2^+^ oligodendroglial cell population for percentage of OPCs (PDGFRα^+^), oligodendrocytes (MBP^+^) and proliferating OPCs (Ki67^+^) and (**b**) of DAPI^+^ cells for percentage of oligodendroglial lineage cells (Olig2^+^), neurons (MAP2^+^) and microglia (CD11b^+^), *n* = 6 wells, mean +/− SEM, Mann-Whitney test, (*) *p* < 0.05, (**) *p* < 0.01, representative of two independent experiments. Data are from 5 different experiments depending on timepoint and marker. FOV = field of view = 1 mm^2^

We previously reported enhanced numbers of MBP^+^ oligodendrocytes in mixed glial cultures treated with Treg-conditioned media [16]. In this study we focused on proportional changes of not only oligodendrocytes, but also OPCs and proliferating OPCs relative to the total oligodendroglial population as a more in-depth and alternative quantitative analysis. Additionally, potential Treg effects on other mixed neuron-glia cell populations were also investigated for the first time. In this study, in agreement with our previous study which reported findings based on total cell numbers and MBP^+^ area, treatment with Treg-conditioned media significantly increased the percentage of MBP^+^ oligodendrocytes and reduced the percentage of PDGFRα^+^ OPCs by approximately 20%, while there was no overall effect on the percentage of proliferating OPCs (see Fig. 2a). There was no effect of Treg-conditioned media on percentage of microglia in mixed glial cultures. The astrocytic layer also did not seem to differ based on morphology. Interestingly however, Treg-treated cultures showed a significant reduction in the percentage of neurons (from approximately 20 to 15%, see Fig. 2b). Morphologically, Treg-treated cultures had fewer dense neuronal cell clusters compared to control conditions and instead more single neurons were detected.

### Enhancing effect of Treg-conditioned media on oligodendrocyte differentiation is not caused by exogenous polarizing factors

Treg were generated from CD4^+^ T cells that were polarized with IL-2, TGF-β and anti-IFN-γ. Harvested Treg-conditioned media could still contain polarizing factors even though most will have been consumed by T cells during the 3 day activation period. Reports of the effects of TGF-β on oligodendrocyte differentiation are conflicting between different studies, showing an inhibition of oligodendrocyte differentiation, inhibition of OPC proliferation or enhancement of OPC proliferation [21–23]. This reported difference could be due to the different models utilized including primary rat or mouse OPCs with different purification methods. However, TGF-β treatment clearly has the potential to influence oligodendrocyte differentiation. IL-2 has also been described in the literature to enhance oligodendrocyte differentiation [24]. Even anti-IFN-γ could potentially affect oligodendrocyte differentiation, as OPCs express the γ-chain of Fc receptors (FcRγ) and signaling through FcRγ has been described to enhance oligodendrocyte differentiation via Fyn kinase [25].

**Fig. 3:**
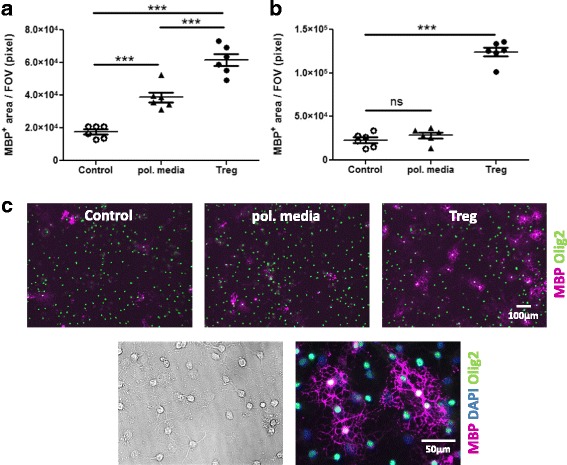
Exogenous Treg-polarizing factors do not directly affect OPC differentiation. (**a**) Mixed neuron-glia cultures were treated with Treg-polarizing media (pol. media), a mixture of TGF-β (300 pg/ml), anti-IFN-γ (500 ng/ml) and IL-2 (500 pg/ml), compared to Treg-conditioned media and matched medium control (X-VIVO™15) for 5 days (**b**) Pure OPC cultures were treated with a mixture of TGF-β (300 pg/ml), anti-IFN-γ (500 ng/ml) and IL-2 (500 pg/ml) compared to Treg-conditioned media and matched medium control (BSM) for 3 days. Immunofluorescence analysis of MBP^+^ area, *n* = 6 wells, mean +/− SEM, unpaired t-test, (***) *p* < 0.001, representative of two independent experiments. FOV = field of view. (**c**) Representative images of pure OPC cultures stained for oligodendrocytes (Olig2^+^MBP^+^) at 10× and 40× magnification at 12 div

To confirm that the enhancement of oligodendrocyte differentiation was indeed mediated by Treg and not an artificial effect due to the use of Treg-polarizing factors, the effects of the individual polarizing factors on MBP^+^ area were investigated. While MBP^+^ area represents a more direct measure of oligodendrocyte myelin protein elaboration, its use as a surrogate marker for Treg-enhanced oligodendrocyte differentiation was confirmed via MBP^+^ cell counting in Treg-enhanced oligodendrocyte differentiation [16]. Individually, none of the factors used to polarize Treg affected oligodendrocyte differentiation in mixed neuron-glia cultures at polarizing factor concentrations equivalent to 5% Treg-polarized media (see Additional file 1: Figure S2A,B). When a mix of all polarizing cytokines was added to mixed neuron-glial cultures there was a significant enhancement of oligodendrocyte differentiation, although still to a lesser extent than Treg-enhanced oligodendrocyte differentiation (see Fig. 3a). It should be noted that the concentrations of polarizing factors used in this experiment, where equivalent to starting concentrations used induce Treg polarization and amounts remaining in conditioned media after 3 days of activation are likely much lower. To exclude a potential direct effect of Treg-polarizing factors on oligodendrocyte differentiation, pure OPC cultures were also treated with a mixture of IL-2, TGF-β and anti-IFN-γ at concentrations equivalent to 5% Treg-polarized media treatment. Treg-enhancement of oligodendrocyte differentiation in pure OPC cultures was not mediated by exogenous Treg-polarizing factors, but by products released by Treg (see Fig. 3b, c). Therefore, while polarizing factors did not directly affect oligodendrocyte differentiation, there appears to be an indirect effect on oligodendrocyte differentiation in mixed neuron-glia cultures at maximum concentrations, and this effect is significantly less than Treg-enhanced oligodendrocyte differentiation.

### Concentration response of Treg-enhanced oligodendrocyte differentiation

**Fig. 4:**
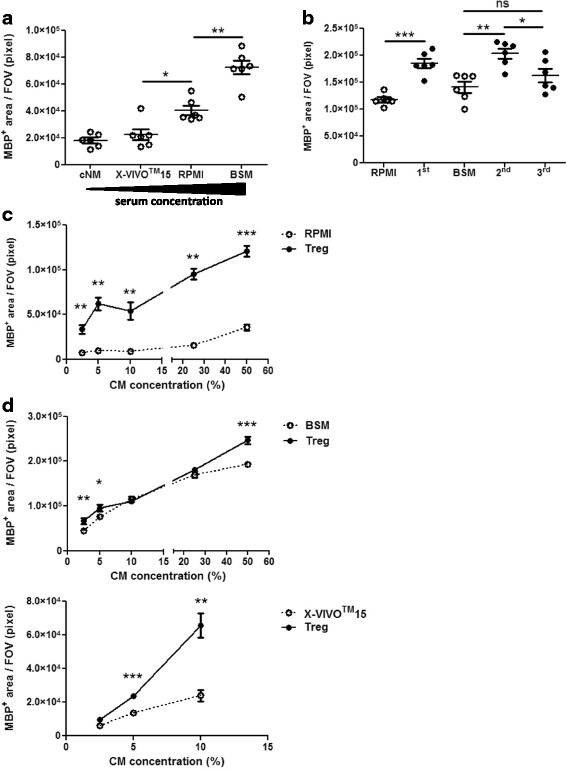
Concentration response of Treg-enhanced OPC differentiation. (**a**) Mixed neuron-glia cultures were treated with 5% of different media used to activate T cells. (**b**) Mixed neuron-glia cultures were treated with 5% of Treg-conditioned media generated from the 1^st^, 2^nd^ and 3^rd^ activation or matched medium control (RPMI for 1^st^ and BSM for 2^nd^ and 3^rd^ activation) for 5 days. (**c**, **d**) Mixed neuron-glia cultures were treated with 2.5, 5, 10, 25 and 50% of Treg-conditioned media from (**c**) 1^st^ and (**d**) 2^nd^ Treg activation or matched medium control (**c**) RPMI, (**d**) BSM or X-VIVO™15 as indicated) for 5 days. (**a**-**d**) Immunofluorescence analysis of MBP^+^ area, *n* = 6 wells and for (**d**) X-VIVO™15 *n* = 5 wells, mean +/− SEM, (**a**) Mann-Whitney test, (**b**-**c**) unpaired t-test, (**d**) unpaired t-test or Mann-Whitney test as appropriate, (*) *p* < 0.05, (**) *p* < 0.01, (***) *p* < 0.001, (**a**-**b**) representative of two independent experiments and (**c**-**d**) data are from a single experiment. FOV = field of view = 1 mm^2^

Different cell types are cultured in varying media by different laboratories. Therefore, determining the differential effect of T cell media on OPC differentiation was critical. Treg were activated multiple times in different media (RPMI for 1^st^ and BSM or X-VIVO™ 15 for subsequent activations) and Treg-conditioned media were found to enhance oligodendrocyte differentiation irrespective of media type. However, the different media types also enhanced oligodendrocyte differentiation to differing degrees. Media containing a higher percentage of serum enhanced oligodendrocyte differentiation more strongly, with X-VIVO™ 15 medium (serum-free), RPMI medium (10% FBS) and BSM (25% horse serum) inducing sequentially increasing oligodendrocyte differentiation (see Fig. 4a). These differences highlight the importance of using appropriate controls, particularly in such neuroimmunological studies in which different media support different cell types.

Treg can be activated multiple times in vitro. This can be useful in producing a higher sample volume from the same number of mice, thereby minimizing animal use. Therefore, the effect of different cycles of T cell activations as well as different media types on Treg-enhanced oligodendrocyte differentiation was investigated. Surprisingly, Treg-conditioned media from both the 1^st^ and 2^nd^, but not the 3^rd^ activation of Treg enhanced oligodendrocyte differentiation (see Fig. 4b). Next, we determined the minimum percentage of Treg-conditioned media necessary to enhance oligodendrocyte differentiation consistently, regardless of the type of conditioned media used. Interestingly, non-linear concentration-dependent effects of Treg-conditioned media were observed, especially when using serum-containing media suggesting rate-limiting steps in downstream signal transduction in oligodendrocyte differentiation. Overall, the lowest concentration of Treg-conditioned media that elicited a significant enhancement of oligodendrocyte differentiation in all tested samples was as low as 5%, which was used in all further experiments (see Fig. 4c, d).

### Timecourse of Treg-enhanced oligodendrocyte differentiation

**Fig. 5:**
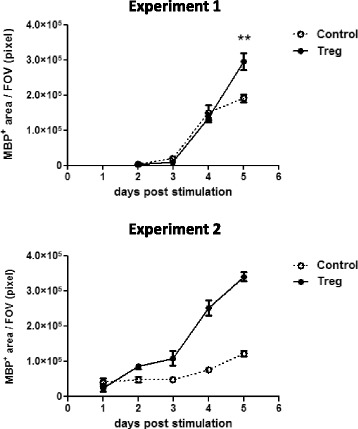
Timecourse analysis of Treg-induced oligodendrocyte differentiation. Mixed neuron-glia cultures were treated with 5% Treg-conditioned media or matched medium control (BSM) for up to 5 days. Immunofluorescence analysis of MBP^+^ area, *n* = 6 wells for Experiment 1 and *n* = 3 wells for Experiment 2, mean +/− SEM, unpaired t-test and Mann-Whitney test as appropriate, (**) *p* < 0.01. FOV = field of view = 1 mm^2^

To ascertain the kinetics of oligodendrocyte differentiation induced by Treg-conditioned media timecourse experiments were performed. Treg-conditioned media consistently enhanced oligodendrocyte differentiation robustly after 5 days of treatment (see Fig. 5) with signs of enhanced differentiation as early as 2 days after treatment in one experiment. For consistency, further experiments investigating the effects of Treg on oligodendrocyte differentiation were all treated for 5 days. These experiments highlight the inherent variability of murine mixed neuron-glia cultures, which is likely caused by the range of pup ages used (from 2 to 7 days), and emphasizes the importance of kinetic studies such as this to determine optimum settings for reliable investigations.

### Treg-conditioned media do not affect OPC proliferation or survival

**Fig. 6:**
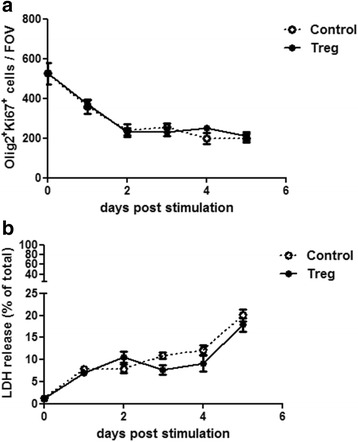
Treg do not affect OPC proliferation or survival in vitro. Mixed neuron-glia cultures were treated with 5% Treg-conditioned media or matched medium control (RPMI) for up to 5 days. (**a**) Immunofluorescence analysis of Olig2^+^Ki67^+^ counts as a timecourse after 1 to 5 days of treatment, *n* = 6 wells, mean +/− SEM, Mann-Whitney test, representative of two independent experiments. FOV = field of view = 1 mm^2^. (**b**) LDH assay of mixed neuron-glia conditioned media after up to 5 days of treatment, *n* = 6 wells, mean +/− SEM, Mann-Whitney test, representative of two independent experiments

While treatment with Treg-conditioned media enhanced the number of differentiated OPCs, this effect could also be due to an underlying effect of Treg-conditioned media on OPC proliferation or survival. Even though we showed that OPC proliferation was unaffected by treatment with Treg-conditioned media after 2 days of treatment [16] this did not rule out an effect at different time-points. Therefore, the effect of Treg-conditioned media on OPC proliferation was investigated at multiple timepoints. As PDGFαα was withdrawn on the day of treatment, maximal OPC proliferation was expected to be earlier than enhancement of oligodendrocyte differentiation, which was observed at 5 days after treatment (see Fig. 5). Indeed, OPC proliferation was highest before PDGFαα-withdrawal and slowly decreased over time. Treg-conditioned media did not affect OPC proliferation at any time-point investigated up to 5 days after treatment (see Fig. 6a).

Even though there was no effect of Treg-conditioned media on OPC proliferation, an enhanced survival of OPCs could still underlie the observed enhancement of oligodendrocyte numbers. We previously showed no effect of Treg-conditioned media on the number of EthD1^+^ cells at one time-point (after 5 days of treatment) [16], therefore the effect of Treg-conditioned media on cell death was investigated at a range of timepoints. There was no effect of Treg-conditioned media on cell survival in mixed neuron-glia cultures up to 5 days after treatment as measured by LDH release (see Fig. 6b).

## Discussion

While many different groups utilize a variety of mixed glial cultures to study glial cell populations, the actual composition, reproducibility and dynamics of these cultures are rarely characterized. To investigate natural mediators in the process of oligodendrocyte differentiation, we established and characterized in detail a murine mixed neuron-glia model. Mixed neuron-glia cultures generated from P2–7 C57BL/6 mouse pups were shown to robustly consist of a full astrocytic monolayer and high proportions of oligodendroglial lineage cells, neurons and microglia, making this an excellent reductionist model to investigate effects on oligodendrocyte differentiation in the presence of all major CNS cell components. While oligodendrocyte differentiation can be investigated efficiently in mixed neuron-glia models, one limitation is that this model does not inform about functional de- or remyelination.

We recently showed that Treg-secreted factors enhance oligodendrocyte differentiation, however this has raised several fundamental questions pertaining to the dynamics of the models used. Here we have addressed key questions pertaining to the effects of different media in which Treg were generated as well as the number of times Treg were activated in vitro, the effect of the factors used to polarize Treg in vitro, the effect of different concentrations of Treg-conditioned media as well the dynamics of the Treg effect on mixed neuron-glia cultures.

Treg-conditioned media enhanced the proportions of mature oligodendrocytes. However, as Treg-polarizing media contains exogenous factors known to affect oligodendrocyte differentiation, we validated that these polarizing factors were not responsible for the observed effects. The maximum concentration of polarizing factors (the amount added at the start of T cell activation) was used to confirm the validity of the observed Treg effect. A mixture of all Treg polarizing factors caused an enhancement of oligodendrocyte differentiation in mixed neuron-glia cultures that was significantly lower than Treg-conditioned media. In pure OPC cultures there was no effect of a mixture of all Treg polarizing factors on oligodendrocyte differentiation. These findings indicate that all polarizing factors together at maximum concentrations are capable of inducing oligodendrocyte differentiation through an indirect mechanism, but are not solely responsible for the observed Treg-enhanced oligodendrocyte differentiation. Interestingly, while Treg-conditioned media generated during the first and second activation enhanced oligodendrocyte differentiation to a similar degree, this was not true for Treg activated for a third time. This finding could give helpful insights into the factors secreted by Treg that confer the oligodendrocyte differentiation enhancing effect of Treg. While the effect of Treg did not depend on the cell culture media type that Treg were activated in, it is crucial to highlight that different media affected baseline oligodendrocyte differentiation to highly varying degrees, likely depending on respective serum content. Additionally, there is an inherent variability of Treg-enhanced oligodendrocyte differentiation in mixed neuron-glia cultures due to the range of mouse pup ages used to generate CNS cultures. This explains why consistent enhancing effects of Treg-conditioned media were observed but there was appreciable variation in fold changes between experiments in this primary culture system. This emphasizes the importance of ensuring that appropriate matched controls are used in such studies.

While Treg-conditioned media significantly enhanced the proportion of mature oligodendrocytes and reduced the proportion of OPCs, there was also a surprising inhibitory effect of Treg-conditioned media on the proportion of neurons in cultures. The cause for these reduced numbers of neurons in mixed glial cultures in vitro remains to be investigated. One possible explanation could be that differentiating oligodendrocytes have high metabolic demands due to production of vast amounts of new membrane. As Treg-conditioned media enhances oligodendrocyte differentiation there would be a higher metabolic demand in these cultures and accordingly, less nutrients available to other cells in the culture, potentially causing the death of vulnerable neurons. Another possible explanation could be that Treg-conditioned media exert a toxic effect on neuronal cells. However, in functional models of myelination such as an OPC-dorsal root ganglion (DRG) neuron co-culture, Treg-conditioned media enhanced myelination [16]. Also, Treg-conditioned media enhanced the proportion of myelinated or remyelinated axons in brain slices ex vivo [16], suggesting that Treg do not critically ablate neurons in models of myelination and remyelination. Nevertheless, further investigation into the effects of Treg on neurons in mixed cultures should be performed.

We recently showed that Treg play a beneficial role in myelin regeneration and enhance oligodendrocyte differentiation [16]. Here we sought to clarify whether the enhancing effect of Treg on oligodendrocyte differentiation may have been partially mediated by underlying effects on either OPC proliferation or survival. We did not observe any effects of Treg-conditioned media on either OPC proliferation or survival at any timepoint up to 5 days after treatment. While LDH release does not specifically inform about oligodendroglial-specific survival, together with the results indicating that there was no effect of Treg on OPC proliferation or overall oligodendroglial cell population, these findings suggest a lack of effect on OPC survival. It would be interesting to further investigate whether Treg-conditioned media might affect OPC survival in challenging conditions such as hypoxia or starvation. A pilot study was performed to investigate the effect of neural medium supplement (Neurobrew-21) withdrawal after 7 div. Surprisingly however, withdrawal of this neural supplement reduced overall cell death and enhanced oligodendrocyte differentiation in mixed glial cultures (see Additional file 1: Figure S3), even though it contains known enhancers of oligodendrocyte differentiation such as Triiodothyronine / Thyroid hormone 3 (T3) and other factors required for oligodendrocyte maintenance such as insulin, sodium selenite, putrescine and D-galactose [26, 27]. Indeed, this pilot study questions the usefulness of using Neurobrew-21 supplement in mixed neuron-glia cultures but this certainly requires further investigation.

We recently identified CCN3 as a Treg-secreted factor capable of enhancing OPC differentiation [16]. However, while the absence of Treg-secreted CCN3 impaired OPC differentiation both in brain slices ex vivo as well as in mixed neuron-glia cultures in vitro [16], Treg-derived CCN3 did not affect oligodendrocyte differentiation in a pure OPC model (data not shown). Therefore, Treg-derived CCN3 did not appear to directly mediate oligodendrocyte differentiation, suggesting that the Treg factor(s) exerting a direct effect on OPC differentiation (see Fig. 3) are indeed different factor(s) and that CCN3 mediates its effect through a different cell type or extracellular matrix component present in mixed neuron-glia cultures. Microglia might indeed be prime candidates to facilitate the oligodendrocyte differentiation-enhancing effect of CCN3, as CCN3 was shown to polarize macrophages towards an M2 phenotype [28] and M2 macrophages and microglia, in turn, enhance oligodendrocyte differentiation [29]. Therefore there are likely other Treg-secreted factors responsible for mediating direct effects on oligodendrocyte differentiation, additional to indirect effects mediated by Treg-derived CCN3, that warrant further investigation in the future.

Murine mixed neuron-glia models are useful tools to study effects on glial cell populations in a model of minimum complexity with all the prevalent cell populations of the central nervous system still present. These in vitro models together with pure glial population models, such as pure OPCs, provide an excellent tool for detailed investigation of basic biology and cellular mechanisms. The use of well-characterized and robust in vitro models across different laboratories is imperative for future identification of novel biological functions. This study provides a robust characterization of a murine neuron-glia model and describes the main cell populations composing this culture at different time-points. Furthermore, we described temporal dynamics of Treg-enhanced oligodendrocyte differentiation, as well as effects of varied activations, media, polarizing factors and concentrations. These detailed investigations will support future cell-based studies of oligodendrocyte differentiation as well as investigations in the influence of immune cells and other mediators on this critical cellular process.

## Additional file

Additional file 1: **Figure S1.** Mixed neuron-glia cultures contain a small proportion of endothelial cells. Representative images of mixed neuron-glia cultures stained for microglia (CD45^+^Iba1^+^), neurons (MAP2^+^NF200^+^), astrocytes (GFAP^+^), OPCs (Olig2^+^ABA5^+^PDGFRα^+^Nkx2.2^+^), oligodendrocytes (Olig2^+^O4^+^APC^+^PLP^+^MBP^+^, endothelial cell (CD31^+^), pericytes (PDGFRβ^+^) and NPCs (Nestin^+^Sox1^+^). **Figure S2.** Single exogenous Treg-polarizing factors do not affect OPC differentiation in mixed neuron-glia cultures. Mixed neuron-glia cultures were treated with (A) IL-2 (500 pg/ml) or (B) TGF-β (300 pg/ml) or anti-IFN-γ (500 ng/ml) compared to Treg-conditioned media and matched medium control (BSM) for 5 days. Immunofluorescence analysis of MBP^+^ area, *n* = 6 wells, mean +/− SEM, unpaired t-test, representative of two independent experiments. FOV = field of view = 1 mm^2^. **Figure S3.** NeuroBrew-21 negatively affects OPC differentiation and cell survival. Mixed neuron-glia cultures were treated with normal differentiation medium (= + NeuroBrew-21) or with differentiation medium without NeuroBrew-21 (= -NeuroBrew-21) for 5 days. Immunofluorescence analysis of EthD-1^+^ cell counts and MBP^+^ area, *n* = 6 wells, mean +/− SEM, unpaired t-test, representative of two independent experiments. FOV = field of view = 1 mm^2^. (PPTX 5757 kb)

## Abbreviations

AWERB: Animal Welfare and Ethical Review Body
BSL1: Bandeira Griffonia Simplicifolia Lectin 1
BSM: Brain slice media
cDMEM: Complete DMEM media
cNM: Complete Neural Medium
CNS: Central nervous system
div: Days in vitro
DRG: Dorsal root ganglion
EAE: Experimental Autoimmune Encephalomyelitis
FcRγ: γ-chain of Fc receptors
FMO: Fluorescence Minus One
FOV: Field of view
GFAP: Glial Fibrillary Acidic Protein
LDH: Lactate dehydrogenase
MAP 2: Microtubule Associated Protein 2
MBP: Myelin basic protein
MS: Multiple Sclerosis
NPCs: Neural progenitor cells
OPC: Oligodendrocyte progenitor cells
PDGFRα: Platelet-derived growth factor receptor α
RT: Room temperature
T3: Triiodothyronine / Thyroid hormone 3
Treg: Regulatory T cells
