# Supplementary material for: Characterization of a murine mixed neuron-glia model and cellular responses to regulatory T cell-derived factors
Source: Mol Brain. 2018 May 2;11:25. doi: 10.1186/s13041-018-0367-6 (PMC5932845; doi:10.1186/s13041-018-0367-6)
Supplement: Supplementary file 1 — Figure S1. Mixed neuron-glia cultures contain a small proportion of endothelial cells. Representative images of mixed neuron-glia cultures stained for microglia (CD45+Iba1+), neurons (MAP2+NF200+), astrocytes (GFAP+), OPCs (Olig2+ABA5+PDGFRα+Nkx2.2+), oligodendrocytes (Olig2+O4+APC+PLP+MBP+, endothelial cell (CD31+), pericytes (PDGFRβ+) and NPCs (Nestin+Sox1+). Figure S2. Single exogenous Treg-polarizing factors do not affect OPC differentiation in mixed neuron-glia cultures. Mixed neuron-glia cultures were treated with (A) IL-2 (500 pg/ml) or (B) TGF-β (300 pg/ml) or anti-IFN-γ (500 ng/ml) compared to Treg-conditioned media and matched medium control (BSM) for 5 days. Immunofluorescence analysis of MBP+ area, n = 6 wells, mean +/− SEM, unpaired t-test, representative of two independent experiments. FOV = field of view = 1 mm2. Figure S3. NeuroBrew-21 negatively affects OPC differentiation and cell survival. Mixed neuron-glia cultures were treated with normal differentiation medium (= + NeuroBrew-21) or with differentiation medium without NeuroBrew-21 (= -NeuroBrew-21) for 5 days. Immunofluorescence analysis of EthD-1+ cell counts and MBP+ area, n = 6 wells, mean +/− SEM, unpaired t-test, representative of two independent experiments. FOV = field of view = 1 mm2. (PPTX 5757 kb) [file 13041_2018_367_MOESM1_ESM.pptx]

## Slide 1
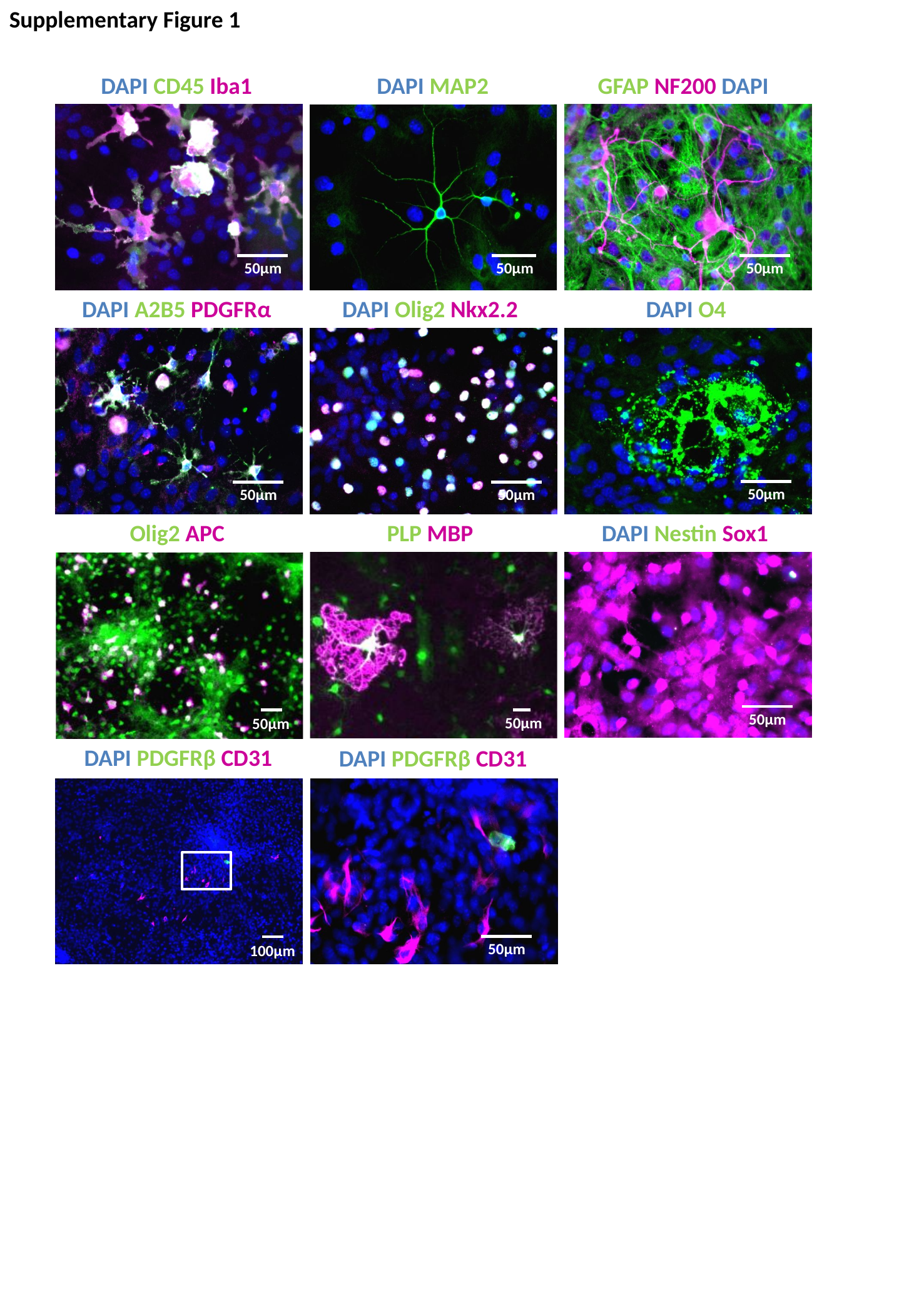

Supplementary Figure 1
DAPI CD45 Iba1
DAPI MAP2
GFAP NF200 DAPI
50µm
50µm
50µm
DAPI A2B5 PDGFRα
DAPI Olig2 Nkx2.2
DAPI O4
50µm
50µm
50µm
Olig2 APC
PLP MBP
DAPI Nestin Sox1
50µm
50µm
50µm
DAPI PDGFRβ CD31
DAPI PDGFRβ CD31
50µm
100µm

## Slide 2
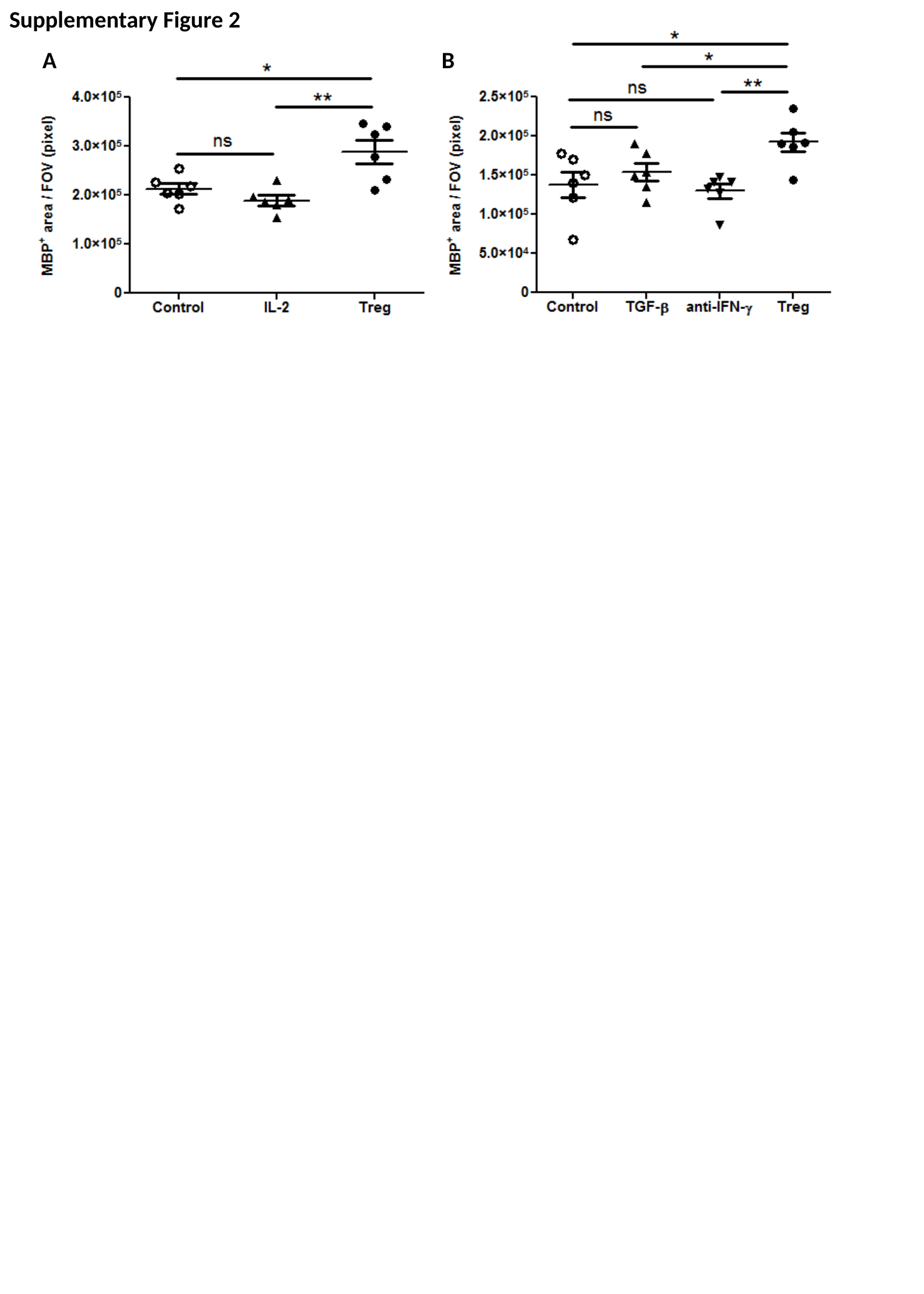

Supplementary Figure 2
A
B

## Slide 3
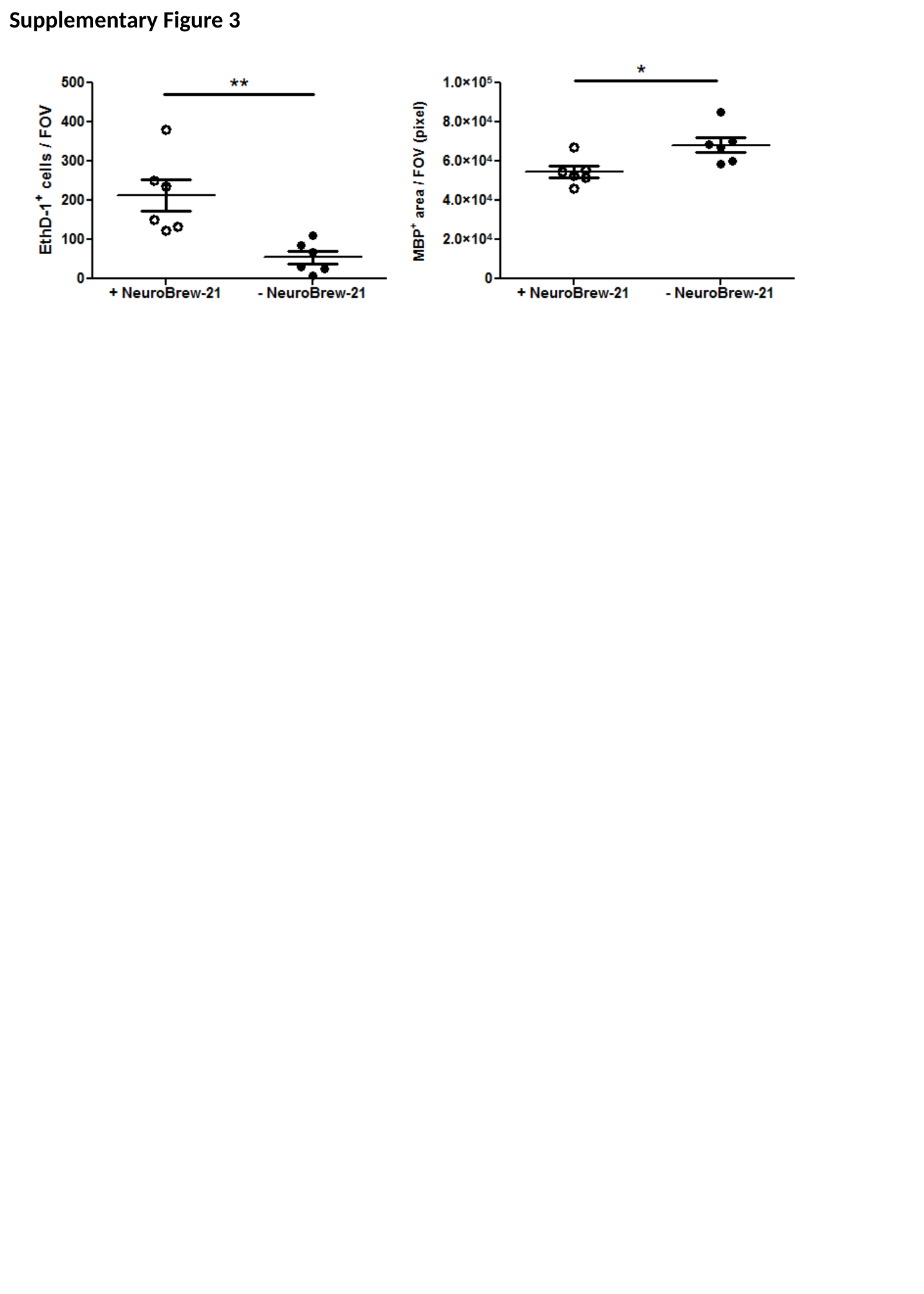

Supplementary Figure 3
